# A Comprehensive Review on Non-Alcoholic Fatty Liver Disease

**DOI:** 10.7759/cureus.50159

**Published:** 2023-12-08

**Authors:** Prerna Sahu, Pratyaksh Chhabra, Ashok M Mehendale

**Affiliations:** 1 Medicine and Surgery, Jawaharlal Nehru Medical College, Datta Meghe Institute of Medical Science, Wardha, IND; 2 Medicine and Surgery, Jawaharlal Nehru Medical College, Datta Meghe Institute of Medical Sciences, Wardha, IND; 3 Preventive Medicine, Jawaharlal Nehru Medical College, Datta Meghe Institute of Medical Sciences, Wardha, IND

**Keywords:** nash, nafld, non-alcoholic fatty liver disease, natural substances, treatment, diagnosis

## Abstract

Non-alcoholic fatty liver disease (NAFLD), now known as metabolic dysfunction-associated liver disease (MASLD), is a spectrum of liver disease. It can be identified by the fact that considerable amount of hepatocytes with minimal or no alcohol use have steatosis*. *Because of its rising incidence along with increasing rates of obesity, metabolic syndromes, and diabetes mellitus type 2, NAFLD is expected to overtake all other causes of cirrhosis over the next decade, necessitating liver transplantation. Nevertheless, heart disease persists as the most prevalent manifestation of mortality, with only a small percentage experiencing fibrosis and complications associated with the liver. Pathologically, NAFLD is linked to lipid toxicity, oxidative stress, lipid deposits, and endoplasmic reticulum stress. A healthy diet, physical exercise, and a decrease in weight are advised by current international guidelines for the treatment of NAFLD, along with a limited number of medicinal therapies, including vitamin E and pioglitazone. Various natural substances have also been identified as NAFLD in vivo and in vitro regulators. The frequency, complexity of the pathophysiology, lack of authorised medications, and difficulty in interpretation of NAFLD have made it a major problem. This article assesses MASLD's pathophysiology, diagnosis, treatment, and epidemiology. This study also reviews a few natural substances that have been shown to have therapeutic advantages for NAFLD.

## Introduction and background

**Figure 1:**
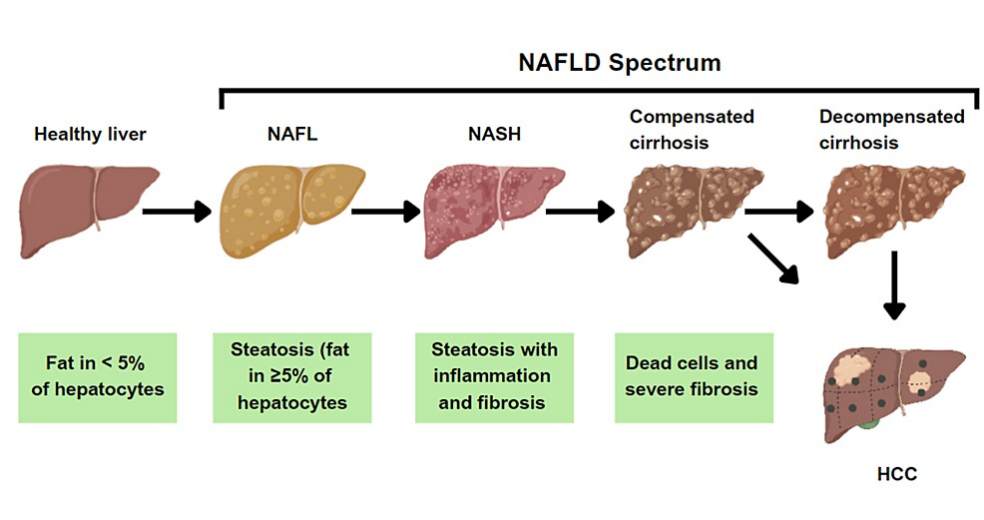
The different phases of NAFLD NAFLD - non-alcoholic fatty liver disease; NAFL - non-alcoholic fatty liver; NASH - non-alcoholic steatohepatitis; HCC - hepatocellular carcinoma Source: [3]

The typical feature of non-alcoholic fatty liver disease (NAFLD), now known as metabolic dysfunction-associated liver disease (MASLD), is microvesicular steatosis in ≥5% of liver cells when there is no underlying etiology, such as alcohol or drug use. It includes a class of illnesses, including non-alcoholic steatohepatitis (NASH), cirrhosis of the liver, non-alcoholic fatty liver (NAFL), and fibrosis [1]. Although NAFLD is presently one of the most prevalent origins of long-term liver disease all over the world, not much is known about the disease, and public discussions about the nation's obesity crisis often ignore the effects of cirrhosis [2]. All patients with steatosis, irrespective of the presence or absence of mild lobular inflammation, are classified as having NAFL. On the other hand, hepatocellular damage is an additional feature of NASH. However, steatosis is known as a "benign" condition, cirrhosis and hepatic cell carcinoma may occur because of its association with liver fibrosis. Figure 1 illustrates the various phases of NAFLD [3]. The estimated global incidence of NAFLD is 47 cases per 1,000 population and is higher among males than females. The estimated global prevalence of NAFLD among adults is 32% and is higher among males (40%) compared to females (26%) [4].

NAFLD can affect skinny, non-diabetic people as well, whereas it is more common in diabetic and obese patients. It is the most common form of idiopathic hepatic disease, and in less than a decade, 50% of NASH patients may develop cirrhosis. It has become more common, along with an increase in obesity and metabolic syndrome, and it is expected to become the main cause of transplantation of the liver within a decade [5].

## Review

Methodology

**Figure 2:**
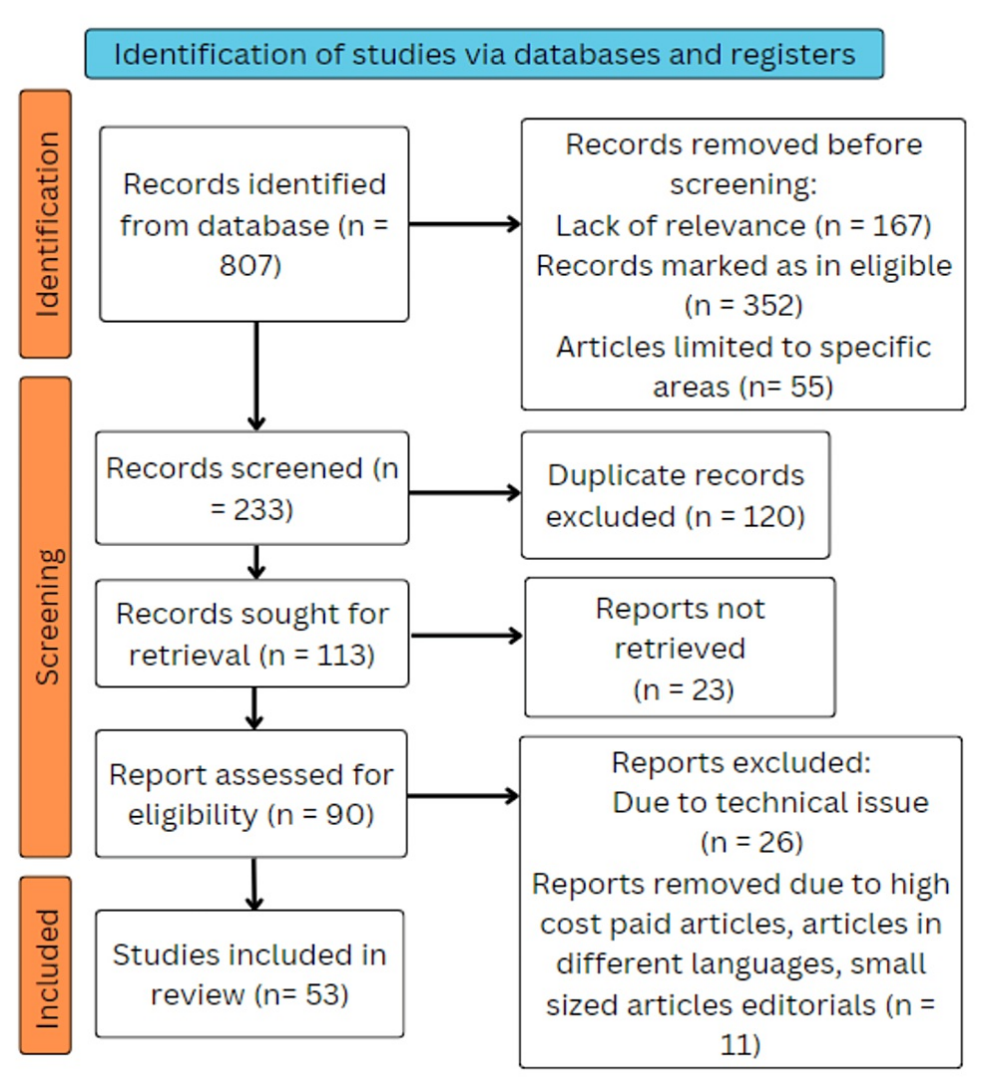
PRISMA framework PRISMA - Preferred Reporting Items for Systematic Reviews and Meta-Analyses

Using the electronic databases PubMed, Medline, Google Scholar, Embase, and ResearchGate, a search of the English-language literature was done. The query terms were "non-alcoholic fatty liver disease" OR "NAFLD"; "pathophysiology" OR "pathogenesis; "diagnostic methods" OR "diagnosis"; "treatment" OR "management"; "natural products" OR "natural ingredients." The following criteria are satisfied by the articles in this review: research focused exclusively on non-alcoholic fatty liver disease (NAFLD), and novel therapeutic approaches were taken into account. We included studies that were done in English from 1998 to 2022. We also searched each bibliography for critical references to pertinent papers. To locate further research, electronic database searches were supplemented with a manual examination of reference lists of relevant publications and review articles. The pathogenesis, diagnosis, and therapy of non-alcoholic fatty liver disease (NAFLD) were examined in observational studies, systematic reviews, experimental studies, and meta-analyses that fit the inclusion criteria. The inclusion of only peer-reviewed and published articles was taken into consideration. Free papers and open-access papers were used. Before incorporating the retrieved papers, two reviewers independently reviewed titles, abstracts, and full texts to make sure they fulfilled the inclusion criteria. Any inconsistencies were resolved through discussion and consensus. The steps for inclusion studies are depicted in Figure 2.

Epidemiology

Globally, the prevalence of NAFLD is estimated to be 25%; it varies from 13% in Africa to 23% in Europe and 32% in the Middle East. Geographic variation is the outcome of differences in disease prevalence and extent that are known to exist among different ethnic groups [6]. Black ethnicity is protective against NAFLD, while Hispanic groups exhibit higher rates of NASH, which might be partially defined by an increased prevalence of genetic risk variants associated with NASH [6]. In Asia, the prevalence of NAFLD is around 30% overall. A meta-analysis of the literature from 2019 included 182 research with 2,385,999 participants, and it showed that 30.5% of Asians were projected to have NAFLD** **[4]**. **Due to the fact that Asia is home to a diverse range of ethnic groups and socioeconomic conditions, the incidence of NAFLD varies greatly across its member nations. With a frequency of 42%, Southeast Asia has the highest rate of NAFLD among Asian sub-regions [4]. NAFLD is directly linked with dyslipidemia, diabetes mellitus type 2, metabolic syndrome, and central obesity, with respective relative prevalence rates of 69%, 23%, 43%, and 51% [7].

As a result, the number of cases of illness rose in tandem with growing obesity rates, rising from 15% in 2005 to 25% in 2010 [7]. Cardiovascular disease accounts for 40% of deaths in people with NAFLD. Current investigations have suggested that NAFLD may independently develop the risk of cardiovascular disease, even if a typical set of risk factors might be equally important [8].

Pathogenesis

**Figure 3:**
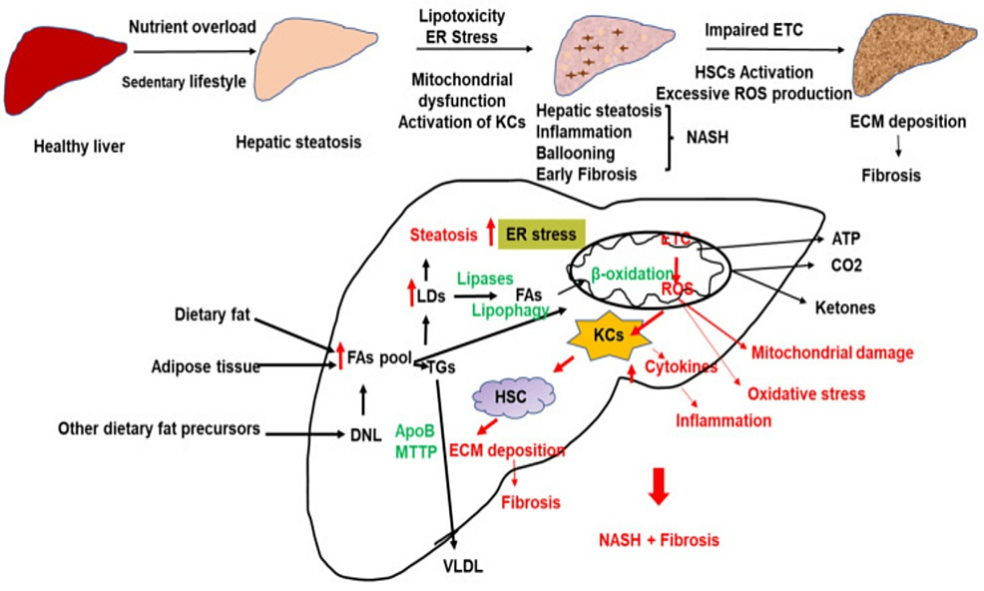
Pathogenic pathways involved in NAFLD NAFLD - non-alcoholic fatty liver disease; KC - Kupffer cells; HSC - hepatic stellate cells; ER stress - endoplasmic reticulum stress; ETC - electron transport chain; NASH - non-alcoholic steatohepatitis; FA - fatty acid; ROS - reactive oxygen species; ATP - adenosine triphosphate; ECM - extracellular matrix; VLDL - very low density lipoprotein; TG - triglycerides; LD - lipid droplets; DNL - De novo lipogenesis; apoB - apolipoprotein B; MTTP - microsomal triglyceride transfer protein Source: [11]

The two-hit theory, which involves several stressors, is still the accepted explanation for how inflammation develops and how NAFLD progresses [9]. The progression of NAFLD is influenced by oxidative stress, endoplasmic reticulum (ER) stress, gut endotoxins, lipid toxicity, mitochondrial dysfunction, and microbiota. Lipid excess can cause fibrosis, oxidative stress, inflammation, and lipid toxicity [9,10]. NAFLD primarily occurs due to a calorie-rich diet, a lack of physical exercise, and the modern way of life. The excess of reactive oxygen species (ROS) may arise from the uncoupling of respiration by adenosine triphosphate (ATP) synthesis due to free fatty acid overload (Figure 3).

Accumulation of Lipid

Lipids are the reserve of extra energy consumed. They are stored throughout the body in different organs when they are disorganized. An example of ectopic lipid buildup is commonly seen in NAFLD. Excessive triglyceride synthesis in hepatocytes is the cause of fatty liver, which occurs in NAFLD; white adipose tissue provides 60% of the substrate for lipid synthesis, de novo lipogenesis provides 26%, and high-fat or high-sugar food intake provides 15% [12]. Insulin stimulates esterification, and the storage of fatty acids inhibits lipolysis and mediates the storage of triglycerides in fat tissue. As a result, insulin resistance (IR) emerges as a crucial component in NAFLD therapy [13]. Fructose-containing diets increased the gene expression linked to endoplasmic reticulum (ER) stress, adipocyte death, inflammation, and liver fibrosis. In contrast, a diet rich in fat by itself induces resistance to insulin, obesity, and moderate steatosis with low inflammation and fibrosis [13,14].

Oxidative Stress

Excess carbohydrates are normally converted to fatty acids by de novo lipogenesis. As a result, triglycerides made of these fatty acids are esterified and then stored in hepatocytes. Triglycerides use β-oxidation to supply the individual with energy when it is low on energy. Numerous factors that contribute to the synthesis of free fatty acids in the liver affect β-oxidation and mitochondrial function, which in turn promote inflammation and oxidative stress [15]. ROS plays an important function as an inflammatory response mediator. Reduced respiratory chain complex activity, decreased ATP production, ultrastructural mitochondrial damage, and microsomal lipid-oxidizing cytochrome P450 enzymes as prooxidants are all present in NAFLD patients [16,17]. The mitochondria, which are vital for fatty acid oxidation and energy synthesis, constitute the main source of ROS in cells. However, mitochondria also release a lot of ROS during these processes. Moreover, the oxidation of fatty deposits by ROS releases lipid peroxides that are harmful to hepatocytes. Lipid peroxides and ROS cause further respiratory chain damage in hepatocytes, which either directly or indirectly results in oxidative damage to the mitochondrial genome. This damage then fuels the creation of additional reactive oxygen species, perpetuating the detrimental chain reaction [17].

Endoplasmic Reticulum (ER) Stress

Protein homeostasis is restored by the defensive mechanism, unfolded protein response (UPR), brought on by endoplasmic reticulum stress. However, when the UPR is unable to maintain cell viability, the proapoptotic ER stress pathway activates cells, subsequently leading to cellular death [18]. Complex sphingolipids and a trace quantity of cholesterol make up the ER membrane. Protein transport and the production of new lipids are aided by the loose packing of lipids in the ER membrane. The primary metabolic route impacted by ER stress is lipogenesis [19]. Activating transcription factor 6 (ATF6), and protein kinase RNA-like ER kinase (PERK) and, inositol-requiring enzyme 1 (IRE1) are the three main ER-resident stress sensors that mediate UPR. These substances attach to glucose-regulated protein 78 (GRP78) and retain its inactive state under normal circumstances [20]. All these pathways have the ability to become active under ER stress circumstances following their dissociation from GRP78, which affects several downstream processes [20].

Lipotoxicity

When lipids and metabolites excessively accumulate in non-adipose tissue, they can cause lipid toxicity, which is harmful and damages the tissue [21]. Hepatocyte damage intensifies, and the illness advances to a more serious condition when the concentration of lipotoxic chemicals in hepatocytes is continuously increased above the capacity of the hepatocyte to transport them [22]. In NAFLD, resistance to insulin causes a significant rise in plasma-free fatty acids, and hepatocytes' fatty acid oxidation excess damages mitochondria, producing copious quantities of reactive oxygen species and inducing endoplasmic reticulum stress, oxidative stress, and inflammatory reactions. Lipotoxic species also produce the same harmful consequences [23].

Lipids are not always lipotoxic. Triglycerides and unsaturated double bonds in free fatty acids, for instance, offer protection against liver damage brought on by lipotoxic substances. Furthermore, it has been shown that while monounsaturated oleic acid is less harmful than saturated free fatty acids like stearic and palmitic acid, it can still encourage the development of hepatic steatosis [24]. Hepatocytes, which make up the majority of hepatic parenchymal cells, are the target of the lipid toxicity process. Nonparenchymal cells, on the other hand, such as Kupffer cells (KC) and hepatic stellate cells (HSC) of the liver, are also crucial to the development of NASH [25]. The primary cell population responsible for fibrogenesis of the liver and the advancement of NASH is HSCs. It has been discovered that lipotoxic chemicals' activation of toll-like receptor 4 enhances fibrotic and inflammatory signalling in HSCs [26].

Diagnosis

The majority of NAFLD patients are diagnosed when high transaminases/aminotransferase enzyme levels are inadvertently discovered. While most individuals have high aminotransferase levels, fibrosis or necro-inflammatory alterations might still be present even with normal values [26,27]. Imaging techniques are not very useful for diagnosing NAFLD. Ultrasonography frequently shows hyperechoic liver in NAFLD patients, although the finding is insufficiently specific or sensitive. Furthermore, it has been demonstrated that the three most used imaging modalities (ultrasonography, computed tomography scan, transient elastography, and magnetic resonance imaging) are unable to differentiate between NASH and different types of NAFLD [28]. To distinguish between steatosis alone and NASH and to confirm a finding of NAFLD, a biopsy of the liver is presently the only procedure available. Additionally, it allows for the evaluation of the disease's severity and could provide prognostic information. However, there are currently no clear criteria or recommendations about who needs a liver biopsy and when. Liver biopsy is further hampered by several important restrictions. Firstly, liver biopsy specimens vary in quality. Moreover, research has demonstrated notable variations in biopsy specimen interpretation across and among observers [29]. Since steatosis and NASH cannot be distinguished using normal procedures (i.e., serological testing and imaging techniques), diagnosing NAFLD is difficult. A liver biopsy is thought to be the gold standard for diagnosing NAFLD and can distinguish between NASH and steatosis. However, because of the higher risk of bleeding and its consequences, regular usage is not advised [27-29]. Table 1 explains several types of diagnostic methods that have been successfully put forward in recent decades [30].

**Table 1:** Diagnostic methods for NAFLD NAFLD - non-alcoholic fatty liver disease; NASH - non-alcoholic steatohepatitis Source: [30]

Diagnostic tools	Technique/principle	Features
Serological tests	Aspartate aminotransferase (AST)	Since some NAFLD patients have normal AST and ALT values, elevated levels are not indicative of NAFLD
	Alanine aminotransferase (ALT)	
	AST/ALT	More than one indicates fibrosis
Imaging techniques	Ultrasonography	Does not distinguish between fatty liver and NASH; sensitive when steatosis is greater than 30% of the liver cells
	Magnetic resonance imaging (MRI), computerized tomographic (CT) scanning	More sensitive than ultrasonography; inadequate capacity to differentiate between NASH and fatty liver; costly
	Transient elastography	Able to diagnose fibrosis, but costly
Liver biopsy	Histological assessment of liver tissues: hepatic lesions like fatty liver, inflammation, and enlargement are graded, and fibrosis is staged.	Gold standard but invasive and may be implicated with complications and sampling variableness; able to detect inflammation and steatosis

Classification

Scoring System by Matteoni

The foundation of Matteoni's system includes fibrosis, ballooning degeneration, inflammation, and Mallory hyaline. Type I (simple fatty liver), type II (steatohepatitis), type III (steatonecrosis), and type IV (steatonecrosis plus either Mallory hyaline or fibrosis) were the four groups into which NAFLD patients were divided. Type I was more benign than the necrotic variants, which were believed to be severe. The potential risk of cirrhosis and mortality due to hepatic disorder is greater in the severe variants' method, which does not account for NAFLD in children, even though it assists in determining individuals who are susceptible to cirrhosis and death due to liver disease [31].

Scoring System by Brunt

The method combines fibrosis into a "stage" and steatosis and steatohepatitis into a "grade" [32]. The percentage of hepatocytes affected determines the grade of steatosis, which ranges from one to three (<33%=1; 33-66%=2; >66%=3). Similar to this, steatohepatitis was rated from one to three (one being mild, two being moderate, and three being severe), but the grading system took into account the degree and kind of ballooning, lobular inflammation, portal inflammation, and steatosis. Conversely, fibrosis was categorized on a one to four scale. The whole range of NAFLD as described by Matteoni's methodology, is not covered by Brunt's system. Furthermore, it was not intended to assess NAFLD in younger individuals [33].

NASH Clinical Research Network (NASH CRN) Scoring System

It developed the NAFLD activity score (NAS) and scoring method in 2005 for use in medical research. This method of scoring was created to take into consideration the entire spectrum of NAFLD lesions. Each category was divided on the basis of the histological traits under consideration. A score was given to each of the main categories. Steatosis (0-3), lobular inflammation (0-3), hepatocellular damage (0-2), fibrosis (0-4), and other abnormalities such as Mallory's hyaline and glycogenated nuclei were among the characteristics that were independently linked to NASH. The NAS is the unweighted sum of the scores for lobular inflammation, steatosis, and liver cell enlargement. Biopsies with a score of less than three were labeled as "not NASH," while a NAS of ≥5 was shown to correspond with the NASH diagnosis. However, not every biopsy sample with NAS ≥5 satisfies the evaluative criteria for definitive NASH, and caution needs to be exercised when determining whether NASH is present or absent [34].

SAF (Steatosis, Activity, and Fibrosis) Scoring System

This method has been suggested recently. When defining NAFL and NASH, the SAF takes liver cell enlargement, lobular inflammation, and steatosis into account. The activity, which runs from zero to four, can be described as the total of the lobular inflammation and liver cell enlargement grades. When there is any level of activity in the fatty liver, it suggests that NAFLD is present. This suggests that steatosis (from one to three) and different degrees of activity are necessary for the characterization of either NAFL or NASH [35].

Other

Clinicopathological ratings have also been used for NAFLD for several purposes, such as predicting advanced forms of NASH and choosing patients who require biopsy. These clinicopathological scores take into consideration several variables, including age, triglycerides, diabetes mellitus, hypertension, platelet count, hyperglycemia, albumin, insulin resistance index, AST/ALT ratio, and body mass index (BMI) [36].

Management

**Figure 4:**
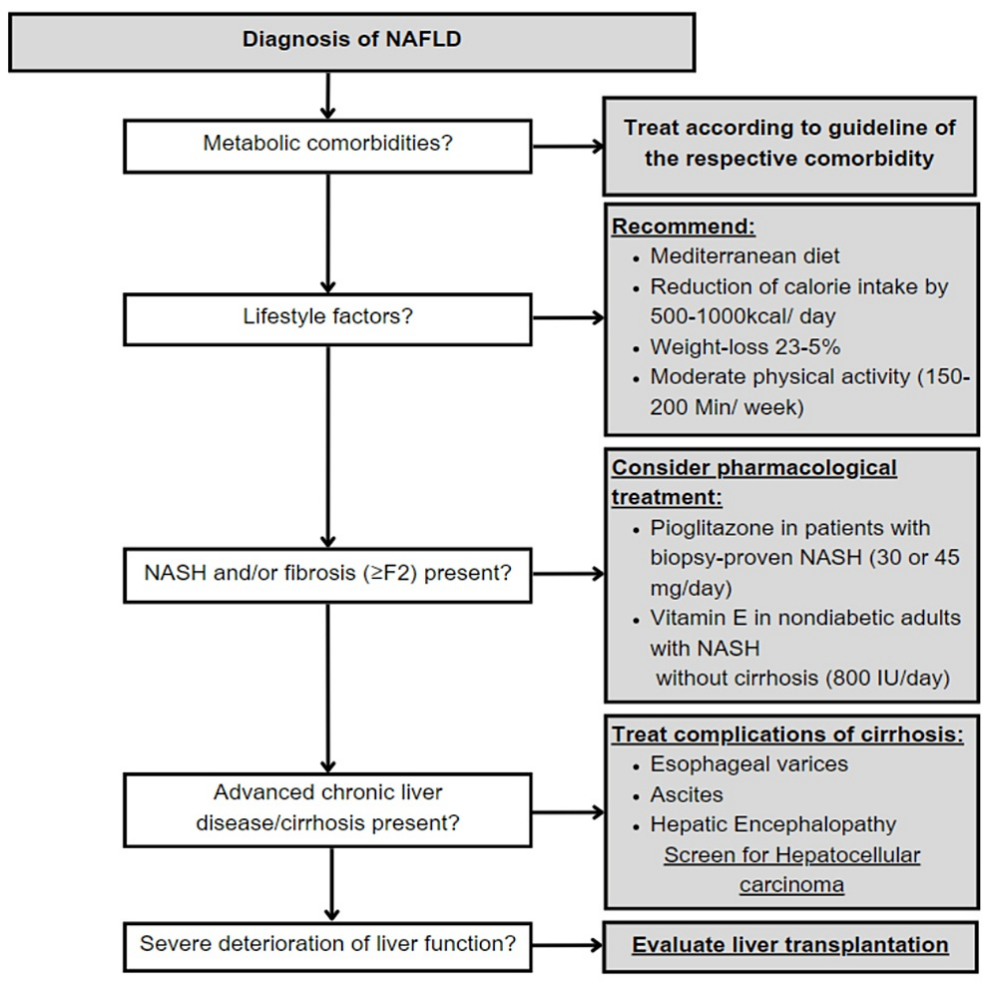
Potential therapy protocol for NAFLD NAFLD - non-alcoholic fatty liver disease; NASH - non-alcoholic steatohepatitis Source: [41]

Current recommendations state that individuals with NASH who have considerable fibrosis (≥F2) or who have a milder form of the disease but possess a high probability of disease development (such as those with diabetes mellitus or metabolic syndrome) should not take medication [37]. However, it is crucial to take into account that individuals with NAFLD have a significantly higher overall death rate than non-NAFLD patients once a diagnosis is made. However, the majority of this increased mortality is due to heart disease rather than liver-related disease [38]; moreover, cancer-associated deaths rank among the most common reasons for death in patients with NAFLD; extrahepatic malignancies lead the way in this regard, followed by hepatocellular carcinoma [39]. Most importantly, individuals are at a higher risk for mortality related to liver disease and complications such as hepatocellular carcinoma, hepatic decompensation, and once a confirmatory diagnosis of NASH and/or advanced fibrosis (cirrhosis, fibrosis stage 3), and/or portal hypertension has been established [40]. Therefore, all individuals with NAFLD should undergo lifestyle adjustments and therapy for underlying metabolic disorders; individuals with biopsy-confirmed NASH and less severe fibrosis should get targeted pharmaceutical treatment. Figure 4 provides a brief synopsis of a potential therapy protocol for NAFLD patients [41].

Lifestyle Factors

Physical exercise, diet, and weight loss are the mainstays of any NAFLD therapy plan. It has been demonstrated that lowering caloric intake by at least 500-1000 kcal can lessen insulin resistance and hepatic steatosis [37]. The EASL-NAFLD guidelines advise low-energy diets and the elimination of NAFLD-promoting foods and goods (processed foods, high-added fructose products), and they typically suggest a "Mediterranean diet" for all NAFLD patients [38]. In the end, dieting results in weight reduction, and weight loss in general has been strongly associated with improving the histology of the liver and even resolving fibrosis, or NASH. Hepatic steatosis and the incidence of NAFLD were dramatically decreased in individuals with diabetes mellitus type 2 who participated in a 12-month lifestyle modification program [42].

According to current standards, moderately strenuous aerobic exercises should be performed in three to five sessions for a total of 150-200 minutes each week. In addition, it should be noted that mild-to-moderate physical activity is risk-free, lowers the level of elevated portal vein blood pressure, and is not related to a higher risk of bleeding from varices or other liver-related decompensation in individuals who have severe persistent liver disease (cirrhosis) [43].

Pharmacological Treatment Options

There are presently only two pharmaceutical therapy options that are indicated by guidelines to individuals with NAFLD: the use of vitamin E and pioglitazone, a proliferator-activated receptor gamma (PPAR-y) ligand [37,38].

Vitamin E

One possible explanation for Vitamin E's encouraging outcomes in randomized studies demonstrating a notable amelioration in NASH is its anti-oxidative impact. The Pioglitazone versus Vitamin E versus Placebo for the Treatment of Nondiabetic Patients with Nonalcoholic Steatohepatitis (PIVENS) experiment, the biggest randomized experiment on vitamin E to date, was released in 2010. Although the pre-established primary study aim was not met by pioglitazone, a PPAR-y ligand (30 mg per day) in the PIVENS trial, the predominant outcome, histology of the liver, had been enhanced in 34% of the group receiving pioglitazone compared to 19% in the group receiving placebo [34]. When compared to a placebo, vitamin E administration led to a substantially greater incidence of NASH improvement [44].

As of 2022, the current procedure guidelines approved by the American Association for the Study of Liver Diseases (AASLD) aver that vitamin E (800 IU per day) "may be considered" for managing non-diabetic individuals suffering from NASH, while the existing (2016) European Association for the Study of the Liver (EASL) guidelines cautiously recommend ("could be used") vitamin E for the management of individuals with NASH and at least appreciable fibrosis [38]. Most notably, vitamin E supplementation is not currently advised for the medical management of cryptogenic liver disease, NAFLD without a biopsy of the liver, NASH cirrhosis, or NASH in diabetic individuals [37].

Pioglitazone

Although the pre-established primary study aim was not met by pioglitazone, a PPAR-y ligand (30 mg per day) in the PIVENS trial, the predominant endpoint, histology of the liver, was improved in 34% of the pioglitazone group compared to 19% in the group receiving placebo [33]. Most remarkably, in 47% of pioglitazone cases and 21% of placebo cases, there was a remission of confirmed NASH. Pioglitazone therapy did not affect fibrosis [44].

The advantages of pioglitazone therapy, which include better sensitivity to insulin and management of diabetes mellitus, are weighed against its side effects, which include increased body weight, retention of fluids, a decrease in bone density, and a potential rise in bladder cancer [5,44].

Other Pharmacological Treatment Options

In addition to vitamin E and pioglitazone, several studies examining the mechanisms underlying various forms of medicine in NAFLD have been released in the past few years, with encouraging outcomes. Nevertheless, none have yet appeared in international or national norms. Sodium-glucose cotransporter-2 (SGLT2) inhibitors, farnesoid X receptor (FXR) agonists, dipeptidyl peptidase IV (DPP-IV) inhibitors, and glucagon-like peptide-1 (GLP1) agonists are a few of them [45]. It has been demonstrated that GLP-1 receptor agonists are useful in lowering liver fat content, liver damage indicators, and body weight. Additionally, a number of studies indicate that these medications may slow the advancement of hepatic fibrosis and help a significant percentage of NASH patients resolve their steatohepatitis [46]. 

Bariatric Surgery

In addition to achieving considerable, long-term loss of weight, bariatric surgery attempts to improve the trajectory of illnesses associated with obesity, including diabetes mellitus type 2, dyslipidemia, hypertension, and obstructive sleep apnea [47]. Additionally, it lowers the risk of cardiovascular conditions, including ischemic stroke and myocardial infarction, as well as overall mortality. The most often done endoscopic surgical procedure globally, among the recently developed techniques, is laparoscopic sleeve gastrectomy (LSG). Another endoscopic method prevalent nowadays is the Roux-en-Y gastric bypass (RYGB) [47].

Bariatric surgery may improve NASH and/or even fibrosis in severely obese patients with NAFLD or NASH. It is possibly due to the substantial prevalence of diabetes type 2 remission that follows bariatric surgery; research studies show that 72.75% of patients had their diabetes under control up to two years after surgery [45]. Additionally, bariatric surgery appears to considerably enhance glycemic management. Furthermore, it is believed that improvements in inflammatory activity and lipid metabolism also have a good effect on the severity of NAFLD. Importantly, however, NASH as a condition itself is not an accepted indication for bariatric surgery [48].

Potential natural ingredients for the treatment of NAFLD

Since there are no existing FDA-approved drugs to manage NAFLD, several types of natural substances may be able to alleviate the symptoms of NAFLD. One general approach to treating NAFLD would be to investigate natural products [3].

A valuable edible fungus endemic to Taiwan, Antrodia cinnamomea, yields a kind of β-glucan that is isolated and processed to produce antrodan. In a high-fat and high-fructose diet mouse model, it has been demonstrated that antrodan reduces the total cholesterol, the plasma levels of malondialdehyde, liver enzymes, triglycerides, insulin, glucose, and uric acid, and increases the levels of adiponectin and leptin [49].

Hesperetin is a citrus flavonoid that is a member of the flavanone family and is widely found in orange, lemon, and grape juice, which are staple foods in both Western and Eastern diets. Hesperetin was able to raise levels of antioxidants and decrease liver toxicity and levels of ROS in a mouse model with a high-fat Western diet. According to this study, hesperetin reduces inflammatory cell infiltration, fibrosis, oxidative stress, and hepatic steatosis [50].

Red wine and grapes are two natural sources of the polyphenol component resveratrol. Resveratrol treatment has been demonstrated to enhance the metabolism of lipids and decrease inflammatory mediator characteristics in the hepatocytes of mice given a diet rich in lipids and NAFL [51].

Milk thistle (Silybum marianum (L.) Gaertn.) fruit and seeds are the source of the flavonoid silibinin. By modifying the c-Jun N-terminal kinase (JNK) pathway, silibinin inhibits NASH. In addition to decreasing fat buildup and encouraging β-oxidation in the liver, it also controlled the activities of oxidase and antioxidant enzymes to lessen oxidative stress [52]. 

Future implications

It is yet unknown if improving hepatic fibrosis will prevent liver-related mortality in NASH patients, despite the fact that it is the most important factor influencing all-cause or liver-related mortality in this population. Globally, the prevalence of diabetes and obesity is sharply rising. Hepatic failure, variceal hemorrhage, and hepatocellular carcinoma are NASH-related liver disorders that will soon account for the majority of liver transplant cases [53]. NASH market size is predicted to reach 49 billion in Japan, the United States of America, and European countries like England, France, Germany, Italy, and Spain by 2027 [53]. A summary of all the articles included in this review is listed in Table 2.

**Table 2:** Summary of the findings from various sources included in this review NAFLD - non-alcoholic fatty liver disease; NASH - non-alcoholic steatohepatitis; NAFL - non-alcoholic fatty liver; HCC - hepatocellular carcinoma; ETC - electron transport chain; ROS - reactive oxygen species; UPR - unfolded protein response; ER - endoplasmic reticulum; KC - Kupffer cells; HSC - hepatic stellate cells; NASH CRN - Nonalcoholic Steatohepatitis Clinical Research Network; SAF - steatosis, activity, and fibrosis; BMI - body mass index; ALT - alanine aminotransferase; AST - aspartate aminotransferase; DPP IV - dipeptidyl peptidase IV; GLP-1 - glucagon-like peptide-1; FXR - farnesoid X receptor; SGLT2 - sodium-glucose cotransporter-2; JNK - c-Jun N-terminal kinase

Authors	Year	Country	Findings
Maurice et al. [1]	2018	UK	NAFLD is microvesicular steatosis in more than 5% of hepatocytes. It is a spectrum of disorders.
Neuschwander-Tetri [2]	2017	USA	NAFLD increases in a parallel manner with the rise in obesity.
Guo et al. [3]	2022	China	Histologically, NAFLD is divided into NAFL and NASH. NASH’s transition to cirrhosis and HCC is associated with fibrosis. Natural ingredients are helpful in alleviating the symptoms of NAFLD.
Teng et al. [4]	2023	Singapore	Prevalence in the world is higher in males than females, and in Asia, prevalence is around 30%.
Powell et al. [5]	2021	Australia	NAFLD has a two-directional association with metabolic syndromes and diabetes mellitus.
Bambha et al. [6]	2012	USA	NAFLD is more prevalent in Hispanic ethnicity than black ethnicity.
Younossi et al. [7]	2016	USA	NAFLD is associated with comorbidities like diabetes mellitus type 2, dyslipidemia, obesity, and metabolic syndrome.
Sinn et al. [8]	2016	South Korea	NAFLD is linked with coronary artery disease, not connected with any cardiovascular or metabolic risk factors, and raises the risk of heart disease.
Takaki et al. [9]	2013	Japan	NAFL and NASH progress further due to the presence of multiple parallel factors.
Karkucinska-Wieckowska et al. [10]	2021	Poland	Numerous factors, like oxidative stress, mitochondrial dysfunction, and lipid accumulation, are associated with the progression of NAFLD.
Nassir et al. [11]	2022	USA	Various pathogenic pathways are involved in NAFLD. Lifestyle plays a major role in the causation of NAFLD.
Liu et al. [12]	2010	China	In NAFLD, the cause of hepatic steatosis is immoderate triglyceride synthesis.
Tanase et al. [13]	2020	Romania	Insulin resistance plays a major role in the causation of NAFLD, both directly and indirectly.
Basaranoglu et al. [14]	2013	Turkey	Fructose consumption causes NAFLD through the activation of genetic pathways.
Chen et al. [15]	2020	China	Fatty acid β-oxidation, a non-ETC source of ROS, seems to create higher ROS in hepatic metabolic disorders.
Robertson et al. [16]	2001	Australia	Prooxidants, which are microsomal lipid-oxidizing cytochrome P450 enzymes, are factors associated in the causation and progression of NAFLD.
Pessayre et al. [17]	2002	France	The progression of NAFL and NASH involves numerous harmful cycles involving the production of ROS, mitochondrial dysfunction, and lipid peroxidation.
Flamment et al. [18]	2010	France	The UPR is associated with endoplasmic reticulum stress, which eventually leads to the progression of NAFLD.
Gentile et al. [19]	2011	USA	Hepatic fat reserves and lipogenesis control are related to the UPR.
Hetz et al. [20]	2013	Chile	Three typical stress sensors found in ERs mediate UPR.
Engin [21]	2017	Turkey	Lipotoxicity is the term used to describe the harmful consequences of lipid buildup in non-adipose tissues.
Branković et al. [22]	2022	Serbia	Increased concentrations of lipotoxic substances progress to NASH.
Svegliati-Baroni et al. [23]	2019	Italy	Lipotoxic species cause mitochondrial damage and subsequently produce ROS, which causes oxidative stress, ER stress, and inflammation.
Ricchi et al. [24]	2009	Italy	Oleic acid is less harmful than palmitic acid and stearic acid but can cause steatosis.
Musso et al. [25]	2018	Italy	Non-parenchymal cells (KCs and HSCs) are associated with the development of NASH.
Younossi et al. [26]	1998	USA	Standardized histologic features in the diagnosis of NAFLD-steatosis, inflammation, liver cell injury, and fibrosis.
Mofrad et al. [27]	2003	USA	NAFLD exists even in the presence of normal aminotransferase levels.
Wieckowska, Feldstein [28]	2008	Canada	Imaging techniques are neither sensitive nor specific in the diagnosis of NAFLD.
Ratziu et al. [29]	2005	France	Biopsy of the liver is currently the best accessible method to confirm NAFLD.
Cobbina, Akhlaghi [30]	2017	USA	Serological evaluation and imaging assessments are not specific or sensitive for diagnosis. For the diagnosis of NAFLD, a biopsy of the liver is the gold standard method.
Matteoni et al. [31]	1999	USA	Matteoni's classification system included four groups (types) into which NAFLD patients were divided.
Angulo et al. [32]	2003	USA	Association of liver fibrosis with NASH.
Brunt et al. [33]	1999	USA	Brunt’s classification method combines fibrosis into a "stage" and steatosis and steatohepatitis into a "grade".
Kleiner et al. [34]	2005	USA	NASH CRN developed the NAFLD activity score (NAS) and scoring system, in which each of the five major categories into which the histological traits under consideration were divided was assigned a score.
Bedossa et al. [35]	2012	France	The SAF system takes ballooning, lobular inflammation, and steatosis into account. The activity, which runs from 0 to 4, is defined as the total of the lobular inflammation and ballooning grades.
Harrison et al. [36]	2003	USA	To grade NAFLD, various clinico-biological ratings such as BMI, AST/ALT ratio, albumin, etc., have also been used.
Chalasani et al. [37]	2018	USA	Medications should not be given to those patients with NASH who have considerable fibrosis or have comorbidities. Lifestyle modifications, i.e., physical exercise, diet changes, and weight loss, are very crucial in any treatment plan.
European Association for the Study of the Liver (EASL) et al. [38]	2016	Switzerland	Patients with NAFLD have a higher mortality rate due to cardiovascular disease than liver-related disease. Vitamin E might be considered for non-diabetic patients and could be used for patients with the least significant fibrosis.
Simon et al. [39]	2021	USA	Cancer-related deaths are the majority in patients with NAFLD and extrahepatic cancer, followed by HCC.
Hagström et al. [40]	2017	Sweden	Increased risk of liver-related mortality in patients with a confirmed diagnosis of NASH or advanced fibrosis.
Paternostro, Trauner [41]	2022	Austria	After diagnosis, lifestyle changes should be made, and treatment for pre-existing metabolic disorders should be given. Patients with NASH and less severe fibrosis should be administered targeted drug therapy.
Lazo et al. [42]	2010	USA	Weight loss reduces hepatic steatosis and the occurrence of NAFLD, especially in patients with diabetes mellitus type 2.
Macías-Rodríguez et al. [43]	2016	Mexico	Mild to moderate exercise lowers the level of portal hypertension and is not linked with an increase in variceal bleeding or hepatic decompensation in patients with chronic liver disease.
Sanyal et al. [44]	2010	USA	Vitamin E and pioglitazone improved steatosis and lobular inflammation, but not fibrosis. Vitamin E was more efficient than pioglitazone.
Trauner and Fuchs [45]	2021	Austria	Drugs like DPP-IV inhibitors, GLP-1 agonists, FXR agonists, and SGLT2 inhibitors might be useful in the treatment of NAFLD.
Nevola et al. [46]	2023	Italy	GLP-1 agonists are useful in weight loss and the treatment of steatohepatitis.
Głuszyńska et al. [47]	2021	Poland	Bariatric surgery is useful in the loss of weight and in the alleviation of many other illnesses like diabetes mellitus type 2, obesity etc.
Laursen et al. [48]	2019	Denmark	Bariatric surgery improves NASH and/or even fibrosis due to the remission of type 2 diabetes mellitus. NASH is not an indication for bariatric surgery.
Chyau et al. [49]	2020	Taiwan	Antrodan reduces total cholesterol, liver enzymes, triglycerides, etc., in a mouse model with a high-fat and high-fructose diet.
Li et al. [50]	2021	China	Hesperetin lowers hepatotoxicity and ROS levels in a mouse model with a high-fat diet.
Andrade et al. [51]	2014	Brazil	Resveratrol enhances lipid metabolism and decreases inflammation in a mouse model with a high-fat diet and NAFL.
Liu et al. [52]	2019	China	Silibinin inhibits NASH by modifying the JNK pathway. It also reduces oxidative stress.
Sumida, Yoneda et al. [53]	2017	Japan	NASH market size to increase by 49 billion in countries like Japan, the USA etc.

## Conclusions

With a broad range of clinical presentations, NAFLD is a highly common hepatic ailment. Although there is an initial elevated risk of cardiovascular events in individuals with NAFLD, certain patients may develop acute fibrosis or possibly cirrhosis, which increases the risk of hepatic decompensation and liver-related deaths. For the management of NAFLD, there are currently few pharmaceutical treatments available; the mainstays of any therapeutic plan are exercise, decreasing weight, and a balanced diet. Pharmaceutical therapies now in use include pioglitazone and vitamin E. Given the growing acceptance of bariatric surgery, prospective research addressing the unresolved questions about the relationship between resistance to insulin, steatosis, and the progression of fibrosis should be made available as soon as possible.
